# A Comparative Study of Seminal Plasma and Sperm Metabolome Profiles in Toraya Buffalo Bulls

**DOI:** 10.1155/vmi/9987127

**Published:** 2025-11-04

**Authors:** Tulus Maulana, Syahruddin Said, Rusli Fidriyanto, Raden Iis Arifiantini, Hasbi Hasbi, Jakaria Jakaria, Asep Gunawan

**Affiliations:** ^1^Graduate School of Animal Production and Technology, Faculty of Animal Science, IPB University, Bogor, Indonesia; ^2^Research Center for Applied Zoology, National Research and Innovation Agency, Bogor, Indonesia; ^3^Division of Veterinary Reproduction and Obstetrics, School of Veterinary Medicine and Biomedical Sciences, IPB University, Bogor, Indonesia; ^4^Department of Animal Production, Faculty of Animal Science, Hasanuddin University, Makassar, Indonesia; ^5^Department of Animal Production and Technology, Faculty of Animal Science, IPB University, Bogor, Indonesia

**Keywords:** GC–MS, metabolomics, seminal plasma, sperm, Toraya buffalo

## Abstract

The objective of this study was to compare the metabolomic profiles of seminal plasma and sperm in Toraya buffalo bulls to identify key metabolites that influence sperm quality and reproductive potential. Semen samples were collected from eight mature Toraya buffalo bulls aged 4–10 years and classified as Saleko and Bonga types. Sperm were separated from seminal plasma and subjected to metabolite extraction, followed by derivatization and gas chromatography–mass spectrometry (GC–MS) analysis. Metabolites were identified and quantified using the NIST library, and statistical analysis was performed with MetaboAnalyst 6.0. This study utilized GC–MS to analyze the metabolomic profiles of seminal plasma and sperm from Toraya buffalo bulls. The analysis detected 10 metabolite groups, including carboxylic acids, fatty acids, and steroids. Metabolite enrichment revealed carboxylic acids as dominant in plasma and fatty acyls in sperm. Principal component analysis (PCA) and partial least squares discriminant analysis (PLS-DA) showed clear separation between seminal plasma and sperm. Key metabolites contributing to the separation were identified via variable importance in projection (VIP) analysis. Pathway enrichment analysis highlighted galactose metabolism in plasma and glyoxylate metabolism in sperm. Significant differences in metabolic pathways, such as citrate cycle and unsaturated fatty acid biosynthesis, were observed between the two samples. In conclusion, this study revealed unique metabolomic profiles in the seminal plasma and sperm of Toraya buffalo using GC–MS. Important metabolites such as creatinine, docosahexaenoic acid (DHA), and *α*-ketoglutaric acid may serve as potential biomarkers.

## 1. Introduction

The Toraya buffalo from South Sulawesi, Indonesia, has been officially recognized as a local breed under Minister of Agriculture Decree No. 2845/Kpts/LB430/8/2012. This buffalo plays a vital role in the agricultural and livestock practices of the Sulawesi community [1]. As an endemic species, the Toraya buffalo offers a unique opportunity to study various factors influencing its reproductive biology in a localized context.

Fertility is a fundamental aspect of reproductive success in livestock, including buffalo, which are crucial to agricultural systems in many countries, particularly Indonesia. Among various local buffalo breeds, the Toraya buffalo holds great potential for enhancing livestock productivity. However, efforts to optimize reproductive performance face challenges, such as low artificial insemination success rates, fluctuations in semen quality, and limited molecular information about the reproduction of Toraya buffalo bulls. Selection of bulls based on semen quality parameters and in-depth molecular analyses is necessary to assess the fertility of breeding and prospective bulls [2].

Semen is a complex fluid composed of sperm and seminal plasma. These components complement each other in determining successful fertilization in males. Seminal plasma contains key components, such as inorganic ions, specific hormones, proteins and peptides, cytokines, enzymes, and extracellular vesicles, which contribute to membrane stability, sperm viability, motility, acrosome reaction, and fertilization processes [3]. Sperm fertility, in particular, is influenced by intrinsic sperm factors and seminal plasma components [4]. A comprehensive understanding of the composition of seminal plasma and sperm is essential to identify the molecular mechanisms supporting reproductive functions.

Metabolomics, the comprehensive study of metabolites within biological systems, has become essential for understanding sperm and seminal plasma physiology. This approach enables the noninvasive detection of phenotypic changes and responses to dietary factors in livestock [5]. This analysis allows small molecules such as amino acids, peptides, fatty acids, and carbohydrates to be identified and quantified in biological secretions, cells, tissues, and organs [6].

Recent research indicates that specific metabolites may serve as fertility biomarkers. For instance, 2-oxoglutaric acid and fructose have shown significant correlations with bull fertility, suggesting the potential for similar studies in buffalo [6, 7] Additionally, taurine and hypotaurine metabolism have been found to decrease in low-fertility bulls [8], Other studies reported distinct metabolite profiles between high- and low-fertility bulls, including 44 unique metabolites in high-fertility bulls, 35 in low-fertility bulls, and 33 metabolites with dysregulated expression between the two groups [8, 9].

These findings highlight the specific roles of metabolites in seminal plasma and sperm. While seminal plasma serves as a protective and nourishing medium for sperm, sperm utilize specific metabolites to meet their energy needs during transport and fertilization. Such studies open new avenues in reproductive research, allowing the identification of metabolite profiles that support sperm function and seminal plasma composition.

This study aims to compare the seminal plasma and sperm metabolomic profiles of Toraya buffalo bulls and identify key metabolites influencing sperm quality and reproductive potential. The study seeks to uncover biomarkers indicative of reproductive capacity by employing untargeted gas chromatography–mass spectrometry (GC–MS)-based metabolomics analysis. The findings of this research are expected to provide a scientific foundation for improving the reproductive programs of Toraya buffalo and other livestock species.

## 2. Materials and Methods

### 2.1. Study Design and Experimental Animals

**Figure 1:**
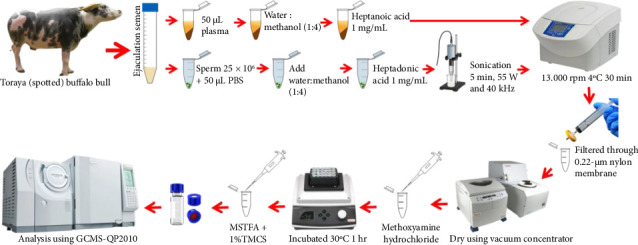
Scheme of metabolomic analysis of Toraya buffalo sperm and seminal plasma.

Semen samples used in the study were obtained from eight Toraya buffalo bulls classified as Saleko and Bonga types. They were four to 10 years old and had an average body of 400–600 kg. The scheme of metabolomic analysis of Toraya buffalo sperm and seminal plasma (Figure 1) and the experimental designs and animal models used in this study were approved by the Animal Ethics Committee of the National Agency for Research and Innovation. Approval was granted under certificate number 050/KE.02/SK/03/2023.

### 2.2. Semen Collection and Preparation

Eight semen samples (one ejaculate per bull) were collected using an artificial vagina, and sperm were separated from seminal plasma by centrifugation (6500 rpm, 4°C, 10 min). Then, the pellet containing sperm was washed twice with phosphate-buffered saline (PBS) and further aliquoted (100 μL) into a new 2-mL Cryotube (Sigma-Aldrich, St Louis, MO, USA). Following the second centrifugation, sperm were snap-frozen in liquid nitrogen and then stored at −80°C until preparation for GC–MS analyses.

### 2.3. Sperm and Seminal Plasma Metabolite Extraction

Sperm and seminal plasma metabolite extraction, as previously described by [10], with modifications and a schematic overview of the sperm metabolite extraction, is presented in Figure 1. The obtained semen plasma was then transferred to 50 μL into a 1.5-mL microtube. Then, 150 μL of heptadecanoic acid in methanol (1 mg/mL, heptanoic acid in methanol as an internal standard; Sigma-Aldrich, St. Louis, USA) and 350 μL of extraction solution (1:4, purified water: methanol) were added to the microtube. The sample was vortexed for one minute and centrifuged at 13,000 rpm at 4°C for 20 min. After centrifugation, the supernatant was filtered (Syringe Filter) and transferred to a 1.5-mL microtube (Agilent Technologies, Santa Clara, CA). The solvent was evaporated to dryness in an evaporator at 36°C for ±2 h. The dried microtubes were stored in the freezer to proceed to the derivatization process.

Sperm pellet was added with 50 μL of saline buffer. Then, 25 μL of the sample was added with 150 μL of heptadecanoic acid in methanol (1 mg/mL, heptanoic acid in methanol as an internal standard; Sigma-Aldrich, St. Louis, USA) and 350 μL of extraction solution (1:4, pure water:methanol) was added to the microtube. The sample was then vortexed, and sperm sonication was performed for 5 min (pulse 10:5) at 25°C using a 55 W and 40 KHz probe sonicator and then centrifuged at 13,000 rpm 4°C for 30 min. After centrifugation, the supernatant was filtered (Syringe Filter) and transferred to a 1.5-mL microtube. The solvent was evaporated to dryness in an evaporator at 36°C for ± 2 h. The dried microtubes were stored in the freezer to proceed to the derivatization process.

The dried extract was then suspended with 50 μL of methoxyamine hydrochloride (20 mg/mL in pyridine; Sigma-Aldrich, St. Louis, USA) and vortexed for 1 min. Then, it was heated in a water bath at 30°C for 1 hour. The samples were then derivatized by adding 100 μL of N-trimethylsilyl-N-methyl trifluoroacetamide with 1% trimethylchlorosilane (MSTFA + 1% TMCS; Sigma-Aldrich, St. Louis, USA) and reheated in a water bath at 70°C for 1 hour. Next, the samples were centrifuged for 10 min at 13,000 RPM and 4°C. The resulting metabolites were transferred to 2-mL amber glass vials with GC vials (Agilent Technologies, Santa Clara, CA). There should be a minimum of 2 quality control (QC) samples, namely, QC1 and QC2, which serve as combined control samples for all test samples. QC samples are prepared by combining equal amounts of each sample to ensure consistent metabolite detection.

### 2.4. GC–MS Analysis

Metabolite analysis of seminal plasma and sperm was conducted using a GC–MS instrument [6, 10] with modification using a different type of machine GCMS-QP2010 (Shimadzu Co., Japan) equipped with an Rtx-5MS capillary column (30 m × 0.25 mm ID × 0.25 μm film thickness; Agilent Technologies). A liquid sample of 1 μL was injected into the GC–MS, with the injector temperature set at 270°C, the interface temperature set at 260°C, and the ion source temperature set at 200°C. Helium (99.9% purity) was used as the carrier gas at a flow rate of 3 mL/min. The oven temperature was initially set at 80°C and held for 2 min, followed by an increase to 325°C at a rate of 10°C/minute, and finally held for 6 min. The solvent cut time was set at 3 min, ionization voltage at 70 eV, and mass range from 30 to 600. The retention index was calculated using alkane standard C8–C20. Compounds were identified by comparing them with the NIST 20 library and the retention index (https://webbook.nist.gov/chemistry/and MS-Search V.3.0 with NIS20 database) [11].

### 2.5. Data Processing, Calculations, and Statistical Analysis

Sperm metabolites were identified by their retention time as well as one target and two quantitative ions, in comparison with mass spectra of authentic standards and mass spectra in the NIST 23 mass spectral library. Abundances of the target ions of metabolites were divided by the abundance of target ion of the internal standard (heptadecanoic acid), and the ratios were used for statistical analysis. Identified compounds were categorized based on their chemical classifications using Human Metabolome Database version 5.0 (HMDB; https://www.hmdb.ca/) [12]. Statistical analysis was carried out using MetaboAnalyst 6.0 Web service (https://www.metaboanalyst.ca). MetaboAnalyst is a comprehensive web-based tool designed to help users easily perform metabolomic data analysis, visualization, and functional interpretation [13]. Sum and auto-scaling normalized each compound. Univariate analysis (*t*-test) was used to determine whether differences in metabolite abundances in seminal plasma and sperm of buffalo bulls were significantly different. Principal component analysis (PCA) retained two components (PC1 and PC2) explaining 81.9% of the total variance. For partial least squares discriminant analysis (PLS-DA), one component was selected based on optimal performance. Model validation was performed using 7-fold cross-validation and 100-permutation testing in MetaboAnalyst 6.0. The PLS-DA model showed high classification performance with one component (accuracy = 0.93, *R*^2^ = 0.91, *Q*^2^ = 0.86), and the permutation test yielded a *p* value of 0.01, confirming the model's statistical validity and low risk of overfitting.

## 3. Results

**Figure 2:**
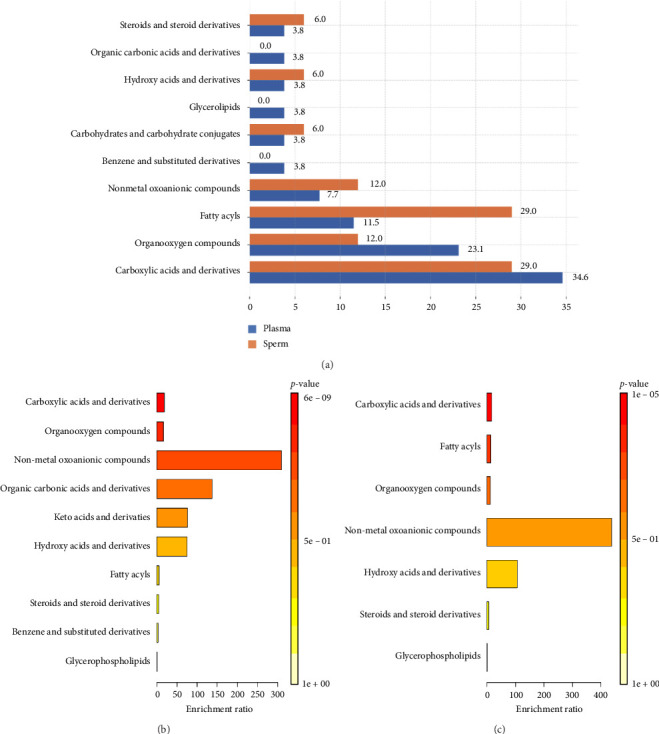
The distribution of metabolites in seminal plasma and sperm (a); metabolite mainclass enrichment overview of Toraya buffalo in seminal plasma (b); and sperm (c).

The GC–MS analysis was performed using GC–MS solution software to process the data. The metabolomic analysis, which utilized GC–MS on both seminal plasma and sperm samples, detected 10 groups of metabolites. These groups include carboxylic acids, benzene, fatty acids, glycerophospholipids, hydroxy acids, keto acids, nonmetallic oxoanions, organic carbonic acids, organooxygen compounds, and steroids (Table 1). The distribution of metabolite compounds in seminal plasma and sperm (Figure 2) showed that carboxylic acids and derivatives are most dominant in seminal plasma (34.6%), while fatty acyls dominate in sperm (29.0%). Organooxygen compounds are higher in plasma (23.1%), whereas nonmetal oxoanionic compounds and benzene derivatives are more abundant in sperm. Some compounds, such as glycerolipids, organic carbonic acids, and benzene and substituted, are only detected in sperm.

Metabolite enrichment analysis results showed that the nonmetal oxoanion compounds group had the highest enrichment ratio and *p* value (*p* > 0.05) in comparison with other metabolite groups in the seminal plasma of Toraya buffalo (Figure 2(b)). In sperm samples, the nonmetal oxoanion compounds group also had a high enrichment ratio value and *p* value (*p* < 0.05) (Figure 2(c)).

**Figure 3:**
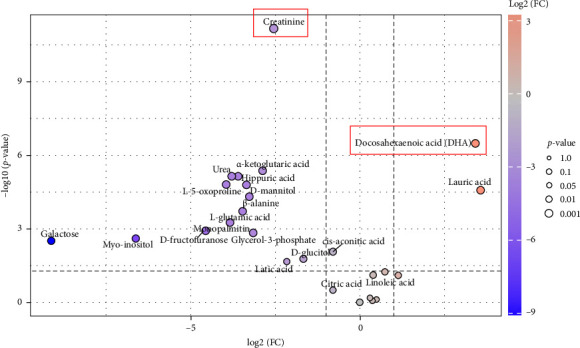
Volcano plot analysis of sperm and seminal plasma Toraya buffalo.

A volcano plot analysis was performed to identify significantly different metabolites between seminal plasma and sperm samples. Several metabolites were found to be differentially expressed based on both fold change and statistical significance (Figure 3). Among the metabolites enriched in seminal plasma were galactose, creatinine, myo-inositol, and urea, whereas sperm samples exhibited higher levels of docosahexaenoic acid (DHA), lauric acid, *α*-ketoglutaric acid (AKG), and L-glutamic acid. These findings are summarized in Table 2, which presents the key differential metabolites along with their log2 fold changes and estimated *p* values.

**Figure 4:**
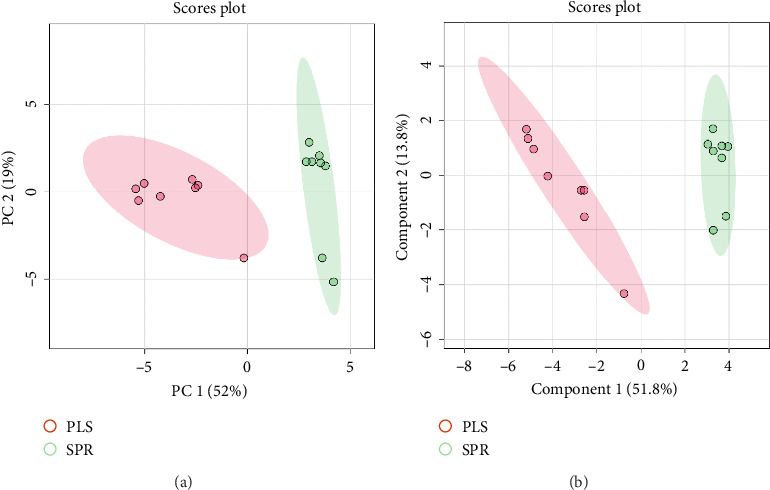
Metabolite profiles of seminal plasma and sperm from Toraya buffalo: (a) principal component analysis (PCA) and (b) partial least squares discriminant analysis (PLS-DA).

**Figure 5:**
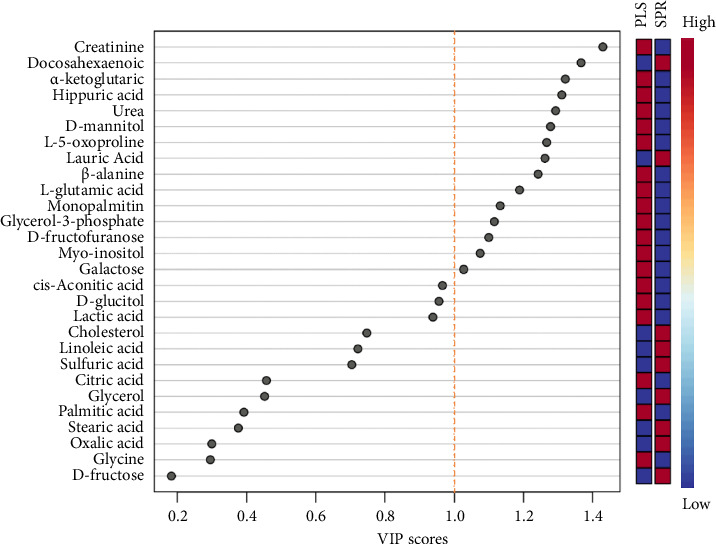
Variable importance in projection (VIP) scores of Toraya buffalo metabolites.

The results of PCA demonstrated the separation of two groups based on metabolite profiles. The *x*-axis represents the first principal component (PC1), accounting for 62% of the variation, while the *y*-axis represents the second principal component (PC2), accounting for 19.9% of the variation. The ellipses on the plot indicate the 95% confidence intervals for each group (Figure 4(a)). PLS-DA results illustrated the separation of seminal plasma and sperm as two distinct groups. The *x*-axis represents the first component (Component 1), explaining 51.8% of the variation, while the *y*-axis represents the second component (Component 2), explaining 13.6% of the variation (Figure 4(b)). Overall, both PCA and PLS-DA plots clearly demonstrated differences in metabolite profiles between seminal plasma and sperm. Variable importance in projection (VIP) analysis identified 15 metabolites with VIP scores greater than 1.0, indicating a significant contribution to the PLS-DA model. These metabolites included creatinine, DHA, AKG acid, hippuric acid, urea, D-mannitol, L-5-oxoproline, lauric acid, *β*-alanine, L-glutamic acid, monopalmitin, glycerol, D-fructofuranose, myo-inositol, and galactose (Figure 5).

The integration of VIP scores with volcano plot analysis revealed several key metabolites that significantly distinguished seminal plasma from sperm samples. Metabolites such as DHA, AKG acid, and L-glutamic acid were significantly enriched in sperm, whereas creatinine, galactose, and myo-inositol were more abundant in seminal plasma. These differential metabolites exhibited both statistical significance (*p* < 0.05) and strong discriminative power (VIP > 1.0). A summary of the top differential metabolites, along with their log2 fold changes, *p* values, and VIP scores, is presented in Table 2.

**Figure 6:**
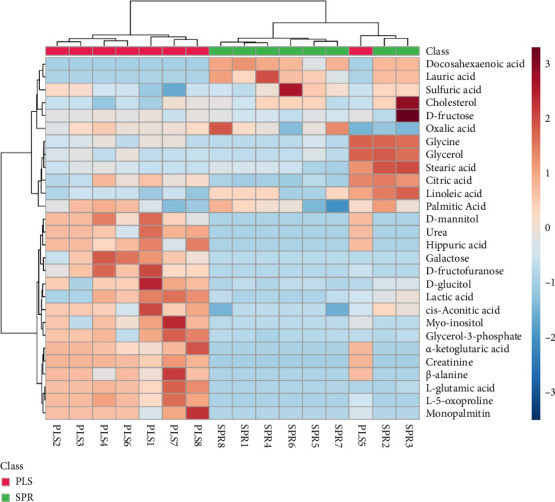
Hierarchical clustering heat map of Toraya buffalo seminal plasma and sperm metabolites.

**Figure 7:**
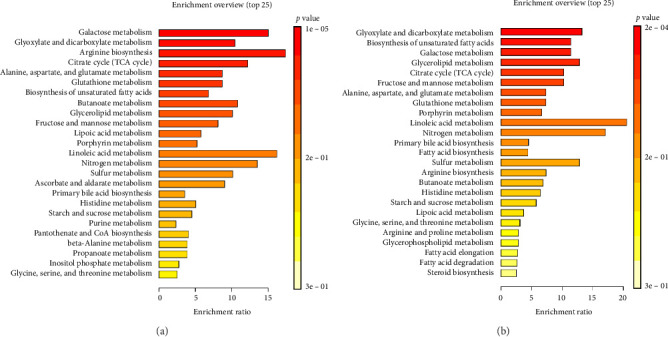
KEGG pathway enrichment analysis of Toraya buffalo metabolites: (a) seminal plasma and (b) sperm.

The dendrogram on the heatmap illustrates the grouping of samples based on metabolite similarity. The results indicate a distinction between samples from the seminal plasma group (PLS) and the sperm group (SPR). However, seminal plasma sample 5 (PLS5) displays a metabolite profile similar to the sperm group (Figure 6). The results of the KEGG (Kyoto Encyclopedia of Genes and Genomes) pathway enrichment analysis for Toraya buffalo metabolites in seminal plasma samples reveal that galactose metabolism exhibits the highest enrichment ratio and most significant *p* value, indicating high activity in seminal plasma. This is followed by glyoxylate and dicarboxylate metabolism, as well as arginine biosynthesis (Figure 7(a)). In sperm samples, glyoxylate and dicarboxylate metabolism also display the highest and highly significant enrichment ratio, similar to seminal plasma (Figure 7(b)).

**Figure 8:**
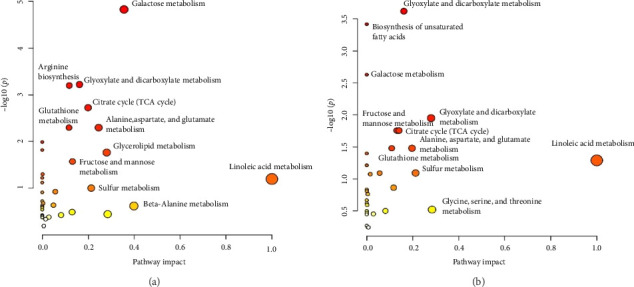
Pathway analysis of Toraya buffalo metabolites: (a) seminal plasma and (b) sperm.

The pathway analysis results include two scatter plots analyzing the metabolic pathways in seminal plasma and sperm. The galactose metabolism pathway exhibits significant importance in seminal plasma (Figure 8(a)), with a −log (*p*) value of approximately 4.82, indicating a substantial impact on plasma. Conversely, in the sperm diagram (Figure 8(b)), the glyoxylate and dicarboxylate metabolism pathway demonstrates high significance and moderate impact, while the biosynthesis of unsaturated fatty acids exhibits significant importance, which is absent in seminal plasma. Although the galactose metabolism pathway remains significant in both samples, it is more predominant in seminal plasma.

Analysis of *p* values and −log (*p*) reveals significant differences between metabolic pathways in both samples (Table 3). The galactose pathway exhibits a *p* value of 0.0001 in plasma (−log (*p*) = 4.824) and 0.0024 in sperm (−log (*p*) = 2.628), indicating high significance in both samples but more pronounced in plasma. The glyoxylate and dicarboxylate metabolism pathway reveals a *p* value of 0.0006 in plasma (−log (*p*) = 3.220) and 0.0002 in sperm (−log (*p*) = 3.619), signifying high significance in both samples. Additionally, the results demonstrate significant differences between plasma and sperm in several pathways. Arginine biosynthesis is highly significant in plasma, with a *p* value of 0.0006 (−log (*p*) = 3.198), but not significant in sperm (*p* value = 0.1373, −log (*p*) = 0.862).

The citrate cycle (tricarboxylic acid [TCA] cycle) also exhibits greater significance in plasma (*p* value = 0.0019, −log (*p*) = 2.724) compared to sperm (*p* value = 0.0174, −log (*p*) = 1.758). In contrast, unsaturated fatty acid biosynthesis displays higher significance in sperm (*p* value = 0.0004, −log (*p*) = 3.416) compared to plasma (*p* value = 0.0103, −log (*p*) = 1.985). Several other metabolic pathways, such as sulfur metabolism and beta-alanine, show low or no significance in both samples, with *p* values of 0.1001 and 0.2427, respectively, and are not detected in sperm.

## 4. Discussion

Characterization of metabolites in seminal plasma and sperm is crucial for understanding the factors influencing sperm quality and fertility. This study utilized untargeted GC–MS-based metabolomic analysis on Toraya buffaloes to identify the primary metabolites in seminal plasma and sperm. The findings revealed that carboxylic acids and their derivatives were the predominant metabolites in both seminal plasma and sperm. Additionally, organooxygen compounds were prominent in seminal plasma, while fatty acids dominated the sperm metabolite profile.

Organooxygen compounds in seminal plasma significantly influence sperm motility through energy provision, membrane stabilization, enzymatic activities, and oxidative stress regulation. The balance of these compounds is crucial for maintaining optimal sperm motility and overall male fertility [7, 14]. Carboxylic acids, organic compounds containing carboxyl groups, play a fundamental role in the structural integrity and functional capabilities of sperm cell membranes and seminal plasma. As a subgroup, fatty acids contribute to forming lipid membranes that determine the physical and chemical properties of sperm membranes [15]. Furthermore, carboxylic acids serve as energy reservoirs supporting sperm motility during transit through the female reproductive tract [16, 17]. Critical reproductive processes, such as the acrosome reaction, also depend on the presence of carboxylic acids [18].

Carboxylic acids are important constituents of seminal plasma, contributing to sperm energy metabolism, protection against microbes, and influencing fertility and the success of sperm cryopreservation. Their specific profiles and concentrations can serve as biomarkers for semen quality and reproductive potential [19, 20]. The concentration and composition of carboxylic acids in seminal plasma significantly influence fertilization outcomes. For example, certain carboxylic acids in pigs are potential biomarkers for sperm cryopreservation ability [21], while seminal plasma proteins interacting with carboxylic acids in cattle enhance sperm motility and fertilization potential [4]. Within sperm, fatty acids contribute to long-term energy metabolism via *β*-oxidation and the TCA cycle, supporting motility and membrane stability.

Specific fatty acids, such as DHA, are critical for maintaining sperm membrane fluidity, protecting against oxidative stress, and enhancing fertilization processes, including the acrosome reaction [16]. Additionally, DHA acts as an antioxidant, safeguarding sperm DNA integrity, promoting testosterone secretion, and supporting spermatogenesis [22]. DHA, a long-chain polyunsaturated fatty acid, is a major constituent of the sperm plasma membrane. It plays a crucial role in maintaining membrane fluidity and flexibility, which are essential for sperm motility, capacitation, and the acrosome reaction [23].

Metabolites with high VIP scores, such as creatinine, DHA, and AKG acid, play essential roles in sperm physiology. Creatinine and urea reflect nitrogen metabolism and oxidative stress levels, while AKG supports energy supply for motility through the TCA cycle and serves as a ligand for oxoglutarate receptor 1 (OXGR1), regulating acid–base balance during sperm maturation [24].

AKG also supports ATP production via the TCA cycle and acts as an antioxidant, reducing ROS-induced sperm damage and preserving sperm viability and function. AKG is an important intermediate in the TCA cycle. It contributes to mitochondrial ATP production and functions as an antioxidant by scavenging reactive oxygen species (ROS), thereby protecting sperm from oxidative stress-induced damage [25].

Creatine plays a crucial role in sperm energy metabolism through the creatine–phosphocreatine system, which supports ATP production and provides an essential energy buffer for maintaining sperm motility and capacitation. By enhancing ATP synthesis via the phosphocreatine shuttle, creatine promotes sperm motility, hyperactivation, and capacitation, partly through the upregulation of ATP availability and tyrosine phosphorylation [26, 27].

Additional metabolites, including L-glutamic acid and D-fructose, are vital for energy production and protein synthesis. L-glutamic acid acts as a precursor in the TCA cycle, while D-fructose serves as the primary glycolytic substrate [9, 10]. Meanwhile, galactose and myo-inositol, both abundant in seminal plasma, contribute to osmotic regulation and membrane stability, which are particularly important during cryopreservation and transport [28].

These metabolites collectively support critical aspects of sperm function, including energy metabolism, membrane integrity, and protection against oxidative stress. Heatmap analysis revealed distinct metabolite distributions between seminal plasma and sperm. Seminal plasma contains glycerol, palmitic acid, and linoleic acid, supporting its nutritional and protective roles. Conversely, sperm is enriched with metabolites such as AKG, creatine, and L-glutamine, which are vital for energy and reproductive functions. These differences underscore the unique physiological adaptations of seminal plasma and sperm. Seminal plasma provides a nutrient-rich, antioxidant-protective environment for sperm, ensuring motility and viability. Proteins and ions such as bicarbonate, calcium, and magnesium regulate critical processes like capacitation and fertilization [29].

Pathway analysis revealed significant differences between the two compartments. Pathways such as galactose metabolism, glyoxylate, and dicarboxylate metabolism were crucial for energy homeostasis and oxidative stress protection. Galactose metabolism was particularly prominent, supporting cryopreservation and sperm health [30]. The TCA cycle, a pivotal pathway in seminal plasma and sperm, drives ATP production, enabling motility, capacitation, and acrosome reactions [31, 32]. Metabolomic analysis of Toraya buffalo semen demonstrated interconnected pathways, each fulfilling distinct roles. Galactose metabolism and arginine biosynthesis are vital in seminal plasma, providing energy and enhancing sperm viability. Galactose serves as an important energy source for sperm. It is present in seminal plasma and can be directly metabolized by sperm cells [33].

The presence and metabolism of galactose and arginine in seminal plasma are critical for optimal sperm function, and they provide essential energy substrates for sperm motility and capacitation [34]. Conversely, sperm prioritizes unsaturated fatty acid biosynthesis and glyoxylate metabolism, ensuring membrane integrity and energy production. During cryopreservation, glycolysis becomes a critical energy pathway for motility, while pathways like glutathione metabolism protect against oxidative stress and maintain DNA integrity.

Pathway enrichment analysis revealed distinct metabolic pathway activations in sperm compared to seminal plasma, reflecting their differing physiological roles. In sperm, pathways such as the TCA cycle, glutamate metabolism, and fatty acid oxidation were significantly enriched. These pathways are directly involved in mitochondrial ATP production, which is essential for sustaining sperm motility, capacitation, and hyperactivation processes that require high energy demand [35].

Conversely, in seminal plasma, enrichment was observed in pathways related to galactose metabolism, inositol phosphate metabolism, and urea cycle. These pathways are associated with osmotic balance, membrane protection, and detoxification, functions that are critical in supporting and preserving sperm viability during ejaculation, transport, and cryopreservation [28]. The presence of these plasma-derived metabolites highlights the compensatory and supportive functions of the seminal plasma microenvironment in safeguarding sperm cells against oxidative stress and physical damage. Accordingly, the observed differences in pathway enrichment reflect the intrinsic metabolic activity of sperm, which is primarily directed toward motility and fertilization capacity, whereas seminal plasma serves as an external medium that facilitates sperm survival, preserves structural integrity, and enhances functional readiness.

This study has several limitations. It was conducted using samples from a single species, the Toraya buffalo (Bubalus bubalis carabanesis), which may limit generalizability to other breeds or species. The untargeted metabolomics approach, while comprehensive, requires further targeted validation. Additionally, the small sample size (*n* = 8) may reduce statistical power. Future studies should involve larger, more diverse populations and include follow-up analyses correlating metabolite levels with sperm quality parameters. Functional validation using IVF assays or sperm function tests is also recommended to confirm the biomarkers' reproductive relevance.

## 5. Conclusion

In conclusion, this study revealed unique metabolomic profiles in the seminal plasma and sperm of Toraya buffalo using GC–MS. Important metabolites such as creatinine, DHA, and AKG acid may serve as potential biomarkers.

## Figures and Tables

**Table 1:** Metabolite classification in Toraya buffalo sperm and seminal plasma.

Group class	Compounds	HMDB ID	RT	RI	RI. ref^a^	SI
Carboxylic acids and derivatives	Creatinine	HMDB0000562	13,653	1574	1548,3	85
	β-Alanine	HMDB0000056	11,735	1437	1439	86
	L-Glutamic acid	HMDB0000148	14,452	1629	1629	94
	Glycine	HMDB0000123	7047	1124	1105	92
	L-5-Oxoproline	HMDB0000267	13,15	1538	1524,2	88
	cis-Aconitic acid	HMDB0000072	16,419	1757	1754	82
	Citric acid	HMDB0000094	17,788	1842	1841	95
	Oxalic acid	HMDB0000094	7418	1147	1145,4	80

Benzene and substituted	Hippuric acid	HMDB0000714	18,008	1856	1853	92

Fatty acyls	Docosahexaenoic acid	HMDB0002183	27,902	2443	2562,2	82
	Lauric acid	HMDB0000638	14,817	1653	1654	85
	Monopalmitin	HMDB0011564	28,127	2456	2606	80
	Palmitic acid	HMDB0000220	21,242	2048	2050	95
	Stearic acid	HMDB0000827	24,541	2244	2250	92
	Linoleic acid	HMDB0000673	24,122	2219	2214	96

Glycerophospholipids	Glycerol	HMDB0000131	9606	1288	1289	95

Hydroxyl acids	Lactic acid	HMDB0000190	6,12	1065	1065	96

Keto acids	α-Ketoglutaric acid	HMDB0302754	15,319	1687	1694	68

Nonmetal oxoanionic	Sulfuric acid	HMDB0001448	7805	1172	1171	92

Organic carbonic acids	Urea	HMDB0000294	8,95	1246	1243	95

Organooxygen compounds	D-Mannitol	HMDB0000765	19,781	1962	1969	93
	Myo-inositol	HMDB0000211	22,584	2128	2129	93
	Galactose	HMDB0033704	18,421	1881	1875	71
	D-Fructofuranose	HMDB0250747	17,581	1830	1792,1	93
	D-Glucitol	HMDB0041500	19,916	1970	1979	95
	D-Fructose	HMDB0000660	17,9	1849	1842	91

Steroids	Cholesterol	HMDB0000067	36,399	2946	3101	82

Abbreviations: RI, retention index; RT, retention time; SI, similarity index.

^a^RI. Ref, Retention index references (https://webbook.nist.gov/chemistry/and MS-Search V.3.0 with NIS20 database).

**Table 2:** Top discriminant metabolites between seminal plasma and sperm based on volcano plot and PLS-DA (VIP score > 1).

Metabolite	Higher in	log2 (FC)	Estimated *p* value	VIP score
Docosahexaenoic acid (DHA)	Sperm	+4.0	< 0.001	1.89
Creatinine	Seminal plasma	−4.5	< 0.001	1.82
α-Ketoglutaric acid	Sperm	+1.8	< 0.01	1.75
Galactose	Seminal plasma	−5.0	< 0.001	1.68
Myo-inositol	Seminal plasma	−4.0	< 0.001	1.60
Lauric acid	Sperm	+3.5	< 0.01	1.58
L-Glutamic acid	Sperm	+1.5	∼0.01	1.54

**Table 3:** Results from pathway analysis of Toraya buffalo seminal plasma and sperm metabolites.

Pathway name	Total compounds	Seminal plasma				Sperm			
		Hits	Raw p	−log (*p*)	Impact	Hits	Raw p	−log (*p*)	Impact
Galactose metabolism	27	5	0.0001^∗^	4.824	0.3561	3	0.0024^∗^	2.628	0
Glyoxylate and dicarboxylate metabolism	32	4	0.0006^∗^	3.220	0.1614	4	0.0002^∗^	3.619	0.1614
Arginine biosynthesis	14	3	0.0006^∗^	3.198	0.1168	1	0.1373	0.862	0.1168
Citrate cycle (TCA cycle)	20	3	0.0019^∗^	2.724	0.1990	2	0.0174^∗^	1.758	0.1404
Glutathione metabolism	28	3	0.0051^∗^	2.295	0.1155	2	0.0330^∗^	1.481	0.1084
Alanine, aspartate, and glutamate metabolism	28	3	0.0051^∗^	2.295	0.2452	2	0.0330^∗^	1.481	0.1971
Biosynthesis of unsaturated fatty acids	36	3	0.0103^∗^	1.985	0	4	0.0004^∗^	3.416	0
Butanoate metabolism	15	2	0.0154^∗^	1.813	0	1	0.1464	0.834	0
Glycerolipid metabolism	16	2	0.0174^∗^	1.758	0.2804	2	0.0113^∗^	1.947	0.2804
Fructose and mannose metabolism	20	2	0.0268^∗^	1.573	0.1308	2	0.0174^∗^	1.758	0.1308
Lipoic acid metabolism	28	2	0.0500	1.301	0.0017	1	0.2568	0.590	0.0017
Porphyrin metabolism	31	2	0.0601	1.221	0	2	0.0399	1.399	0
Linoleic acid metabolism	5	1	0.0637	1.196	1.0000	1	0.0512	1.290	1.0000
Nitrogen metabolism	6	1	0.0760	1.119	0	1	0.0612	1.213	0
Sulfur metabolism	8	1	0.1001	1.000	0.2128	1	0.0808	1.093	0.2128
Primary bile acid biosynthesis	46	2	0.1192	0.924	0.0556	2	0.0811	1.091	0.0556
Ascorbate and aldarate metabolism	10	1	0.1236	0.908	0	—	—	—	—
Histidine metabolism	16	1	0.1906	0.720	0	1	0.1554	0.808	0
Starch and sucrose metabolism	18	1	0.2118	0.674	0.0046	1	0.1732	0.762	0.0046
Pantothenate and CoA biosynthesis	20	1	0.2325	0.634	0.0476	—	—	—	—
Purine metabolism	71	2	0.2366	0.626	0	1	0.5340	0.272	0
Beta-alanine metabolism	21	1	0.2427	0.615	0.3993	—	—	—	—
Propanoate metabolism	22	1	0.2527	0.597	0	—	—	—	—
Pyruvate metabolism	23	1	0.2626	0.581	0	1	0.2160	0.665	0
Gluconeogenesis	26	1	0.2916	0.535	0	1	0.2407	0.618	0
Inositol phosphate metabolism	30	1	0.3285	0.483	0.1294	—	—	—	—
Glycine serine threonine metabolism	34	1	0.3637	0.439	0.2846	1	0.3031	0.518	0.2846
Arginine and proline metabolism	36	1	0.3805	0.420	0	1	0.3179	0.498	0
Glycerophospholipid metabolism	36	1	0.3805	0.420	0.0809	1	0.3179	0.498	0.0809
Pyrimidine metabolism	38	1	0.3970	0.401	0	—	—	—	—
Fatty acid elongation	39	1	0.4051	0.392	0	1	0.3396	0.469	0
Steroid biosynthesis	41	1	0.4209	0.376	0.0284	1	0.3537	0.451	0.0284
Amino and nucleotide sugar metabolism	42	1	0.4287	0.368	0	1	0.3606	0.443	0
Fatty acid biosynthesis	47	1	0.4661	0.332	0.0147	2	0.0842	1.075	0.0147
Steroid hormone biosynthesis	78	1	0.6510	0.186	0.0067	1	0.5687	0.245	0.0067

*Note:* Hits = number of compounds from the sample data that match the compounds in the pathway; Raw *p* value determines the statistical significance of the observed results, smaller *p* values indicate more significant results −log (*p*) value = larger −log (*p*) values indicate higher significance.

^∗^ *p* < 0.05.

## Data Availability

The data supporting the conclusions of this research can be obtained from the corresponding author upon a reasonable request.
